# Exploration in the Mechanism of Kaempferol for the Treatment of Gastric Cancer Based on Network Pharmacology

**DOI:** 10.1155/2020/5891016

**Published:** 2020-10-21

**Authors:** Liangjun Yang, Haiwen Li, Maoyi Yang, Weijian Zhang, Mianli Li, Yifei Xu, Jingwei Li, Jianyuan Kang, Jingchao Zhang, Shaoju Guo

**Affiliations:** ^1^Department of Gastroenterology, Tongde Hospital of Zhejiang Province, Hangzhou 310012, China; ^2^Shenzhen Traditional Chinese Medicine Hospital, Shenzhen 518033, China; ^3^Hospital of Chengdu University of Traditional Chinese Medicine, Chengdu 610075, China; ^4^The Fourth Clinical Medical College of Guangzhou University of Chinese Medicine, Shenzhen 518033, China

## Abstract

**Background:**

Kaempferol is a natural polyphenol in lots of Chinese herbs, which has shown promising treatment for gastric cancer (GC). However, the molecular mechanisms of its action have not been systematically revealed yet. In this work, a network pharmacology approach was used to elucidate the potential mechanisms of kaempferol in the treatment of GC.

**Methods:**

The kaempferol was input into the PharmMapper and SwissTargetPrediction database to get its targets, and the targets of GC were obtained by retrieving the Online Mendelian Inheritance in Man (OMIM) database, MalaCards database, Therapeutic Target Database (TTD), and Coolgen database. The molecular docking was performed to assess the interactions between kaempferol and these targets. Next, the overlap targets of kaempferol and GC were identified for GO and KEGG enrichment analyses. Afterward, a protein-protein interaction (PPI) network was constructed to get the hub targets, and the expression and overall survival analysis of the hub target were investigated. Finally, the overall survival (OS) analysis of hub targets was performed using the Kaplan-Meier Plotter online tool.

**Results:**

A total of 990 genes related to GC and 10 overlapping genes were determined through matching the 24 potential targets of kaempferol with disease-associated genes. The result of molecular docking indicated that kaempferol can bind with these hub targets with good binding scores. These targets were further mapped to 140 GO biological process terms and 11 remarkable pathways. In the PPI network analysis, 3 key targets were identified, including ESR1, EGFR, and SRC. The mRNA and protein expression levels of EGFR and SRC were obviously higher in GC tissues. High expression of these targets was related to poor OS in GC patients.

**Conclusions:**

This study provided a novel approach to reveal the therapeutic mechanisms of kaempferol on GC, which will ease the future clinical application of kaempferol in the treatment of GC.

## 1. Background

As the fifth common malignancy in the world, gastric cancer (GC) is still one of the most common health issues with a significant mortality rate worldwide [1]. Despite the decline in incidence, GC remains the focus of clinical, epidemiological, and translational research. The increase in GC incidence will have substantial economic and social influence and will simultaneously bring about great challenges to healthcare systems all over the world [2]. Although treatments like surgery, radiotherapy, chemotherapy, targeted therapy, and gene therapy have been used to cure GC, the 5-year survival rate was still less than 30% [3]. Therefore, finding new drugs is of great significance for the treatment of GC.

Kaempferol is widely distributed in different plant genera. As a natural flavonoid, kaempferol has a wide range of pharmacological activities, including antioxidant, anti-inflammatory, and antiestrogenic activities [4, 5]. According to the RO5 in PubChem (https://pubchem.ncbi.nlm.nih.gov/compound/Kaempferol) and an experiment conducted in healthy humans, kaempferol showed good oral bioavailability [6]. Specifically, kaempferol was reported to have anticancer properties against various human cancers, including GC [7, 8]. According to a case-control study in Spain, the intake of kaempferol appeared to decrease the risk of GC [9]. An experimental study in vivo has demonstrated that kaempferol can significantly inhibit the growth of GC tumor xenografts [10]. Additionally, kaempferol exerted antineoplastic function through inhibiting proliferation and metastasis, inducing cell cycle arrest and promoting apoptosis and autophagic cell death [8, 10, 11]. However, the molecular mechanisms underlying kaempferol in the treatment of GC have not been fully revealed.

With the deepening understanding of the mechanism of drug action, it is well-recognized that drugs work by regulating multiple proteins rather than a single target.

As a brand-new area of pharmacology, network pharmacology provides new approaches for drug discovery for complex diseases and offers new methods in understanding the mechanism of multiple actions of drugs [12]. To further explore the possible mechanism of action of kaempferol in the treatment of GC, network pharmacology was performed to elucidate the potential mechanism comprehensively. The result of this work will provide potential therapeutic targets for further clinic and basis research.

## 2. Materials and Methods

### 2.1. Predicting Targets of Kaempferol

To obtain the targets of kaempferol, the PharmMapper (http://lilab.ecust.edu.cn/pharmmapper/) and SwissTargetPrediction databases (http://www.swisstargetprediction.ch/) were used. The PharmMapper online tool is an online database for potential drug target identification by matching the query compound to the internal pharmacophore model database via a reverse pharmacophore [13]. SwissTargetPrediction is a web server that predicts the most probable protein targets of small molecules based on a combination of 2D and 3D similarity measures with known ligands [14, 15]. The 3D molecular structure file and the canonical SMILES of kaempferol were imported into the PharmMapper and SwissTargetPrediction databases, respectively. Next, the name of these identified candidate targets was sent to the UniProt database (http://www.uniprot.org/) for normalization.

### 2.2. Collecting Targets Related to GC

To obtain the disease-related genes comprehensively, the GC-related genes were collected from four public database sources, including the Online Mendelian Inheritance in Man (OMIM) database (http://www.omim.org), MalaCards database (https://www.malacards.org/), Therapeutic Target Database (TTD, http://bidd.nus.edu.sg/group/cjttd/), and Coolgen database (http://ci.smu.edu.cn/CooLGeN/). In the Coolgen database, targets with hit scores greater than 5 were selected as the GC-related genes [16]. Finally, GC-related targets were obtained.

### 2.3. Molecular Docking

To gain an insight into the relationship between the candidate proteins and ligand at the molecular level, molecular docking was conducted to assess the strength and mode of interactions between kaempferol and the targets. The docking simulation was conducted by CB-Dock (http://cao.labshare.cn/cb-dock/), a new blind docking method based on cavity detection. It can automatically identify binding sites of a given protein, calculate the center and size, and customize the docking box size according to the query ligands and then perform the molecular docking with a popular docking program, AutoDock Vina [17]. The crystal structures of these targets were downloaded from the protein data bank (http://www.rcsb.org). And the 3D structure of kaempferol was download from the PubChem Compound database (https://pubchem.ncbi.nlm.nih.gov/). Then, the protein and the kaempferol were uploaded to CB-Dock to dock.

### 2.4. GO and KEGG Pathway Enrichment

The Gene Ontology (GO) provides comprehensive information for functional genomics and defines the concepts relating to gene functions [18]. The Kyoto Encyclopedia of Genes and Genomes (KEGG) is a database that is famous for its pathway information [19]. To investigate the biological effects of the kaempferol, GO and KEGG pathway enrichment analyses were conducted and calculated by the Comparative Toxicogenomics Database (CTD; http://ctdbase.org/), which is a robust, publicly available database integrated with functional and pathway data [20]. The enriched GO terms and pathways having a corrected *P* value of less than 0.01 were selected and subjected to further analyses. The subsequent pathways related to GC were picked out based on the pathological and clinical data.

### 2.5. Protein-Protein Interaction Analysis

PPI is fundamental for most biological processes in a living cell and is crucial for understanding cell physiology in normal and disease states. In this work, PPI network mapping was performed on obtained bioactive ingredients and disease targets using the Retrieval of Interacting Genes database (http://string-db.org/; version 10.5) with the species limited to “homo sapiens” and a confidence score > 0.4.

The PPI networks were constructed by Cytoscape (version 3.6.1), a bioinformatics software used for data visualization and integration [21]. To find the highly interconnected regions within the PPI network, the Cytoscape plugin cytoHubba (version 0.1) was used [22]. The hub targets were ranked according to the maximal clique centrality, which has a better performance in the PPI network [23].

### 2.6. Expression Analysis of Hub Targets

UALCAN (http://ualcan.path.uab.edu/analysis.html) is an interactive web portal to analyze The Cancer Genome Atlas (TCGA) gene expression data deeply [24]. In this work, the UALCAN database was used to compare the expression level of hub genes between normal gastric tissue and GC.

To explore the expression of these key targets at the protein level, the immunohistochemistry results on the expression of the histone family proteins in GC were retrieved from the Human Protein Atlas (HPA) database [25].

### 2.7. Overall Survival Analysis of Hub Genes

To explore the hub targets' influence on the overall survival (OS) of GC, a cancer genomics dataset named Kaplan-Meier Plotter (http://kmplot.com/analysis/index.php?p=service) [26], which is capable of assessing the effect of genes on survival, was used to estimate the prognostic significance of each hub gene. The patients with GC were divided into the high and low expression groups, and the two groups were compared by a Kaplan-Meier survival plot. The hazard ratio (HR) with 95% confidence intervals and the logrank *P* value were calculated.

## 3. Results

### 3.1. Target Identification and Analysis

It is a feature that a drug usually binds to multiple targets and molecular targets are involved in multiple processes [27]. Thus, identifying kaempferol's targets is of great significance to understand the molecular mechanisms in the GC therapy. In this work, the PharmMapper and SwissTargetPrediction databases were used to predict the targets of kaempferol. We obtained the top 100 potential human protein targets from the PharmMapper and 103 targets from the SwissTargetPrediction database. After merging the data, 24 duplicated targets for kaempferol were saved (Supplementary Table 1).

**Figure 1:**
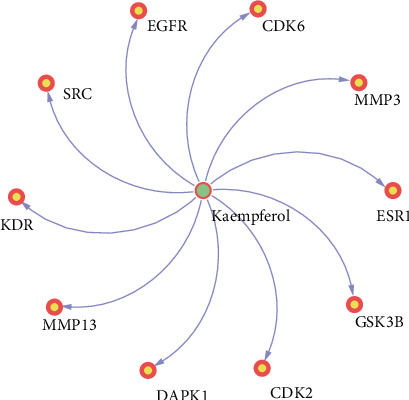
Compound-target network.

By means of the OMIM, TTD, MalaCards, and Coolgen databases, we obtained 990 GC-related targets (shown in Table S2). From an intersection from the two categories of targets, 10 overlapping protein targets were recognized as the targets in the GC treatment (shown in Table 1). Finally, a compound-target network (C-T network) was built. The kaempferol, the targets, and the interactions between them are presented in Figure 1, which have 11 nodes and 10 edges.

### 3.2. Confirmation of Hub Target by Molecular Docking

**Figure 2:**
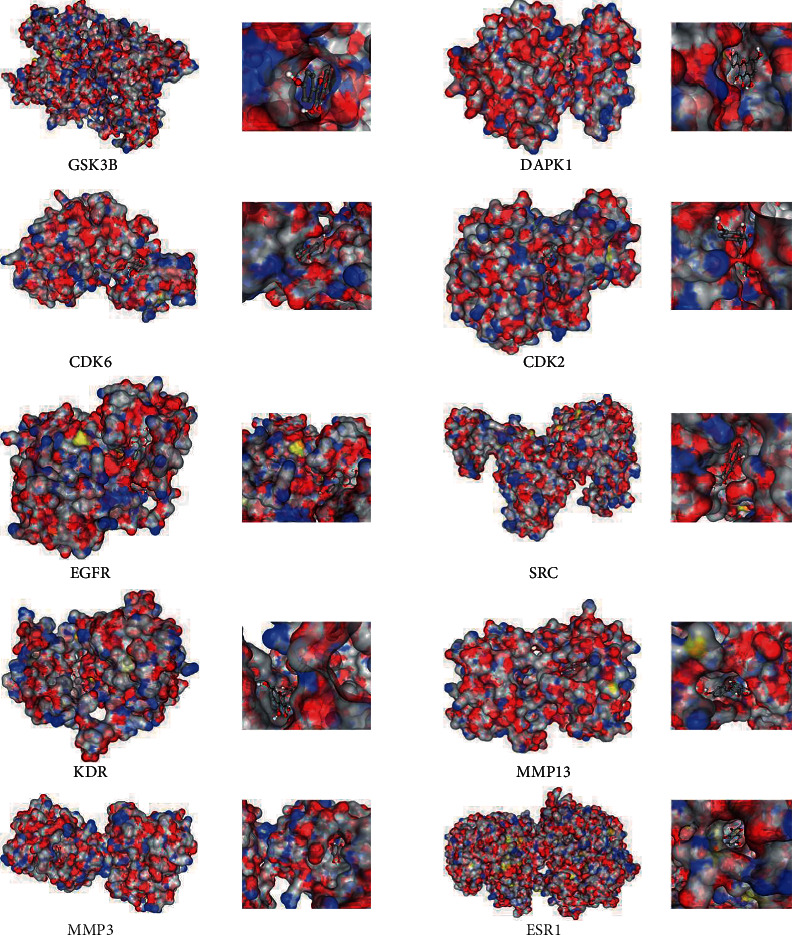
Docking results of kaempferol with the targets.

To verify the reliability of the protein-ligand interactions, 10 proteins were selected as the target for molecular docking based on the predicted results. The structure of kaempferol was upload to CB-Dock for analysis of the docking potential with GSK3B, DAPK1, CDK6, CDK2, EGFR, SRC, KDR, MMP13, MMP3, and ESR1. The docking scores for each target protein are shown in Table 2. According to the Vina score, the result showed that there was a strong interaction between kaempferol and the 10 proteins, which suggested the activity of kaempferol in the treatment of GC. All the docking sketch maps of target proteins with kaempferol are shown in Figure 2.

The crystal structure of the protein active site is colored white (carbon), red (oxygen), blue (nitrogen), and yellow (sulfur). The crystal pose of the ligand is colored white (hydrogen), grey (carbon), and red (oxygen).

### 3.3. GO and KEGG Pathway Enrichment Analyses

**Figure 3:**
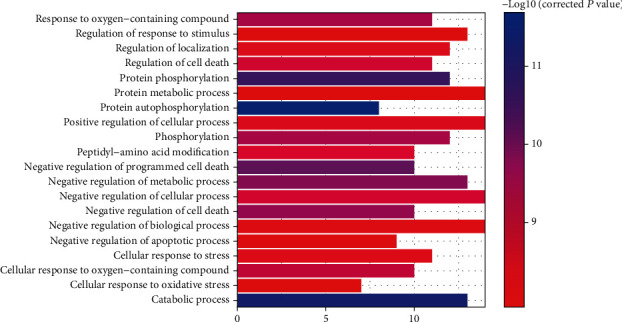
GO analysis of target genes.

To understand the biological processes for kaempferol against GC, GO analysis of 10 candidate targets was performed using the CTD database. According to enrichment results, these targets were significantly assigned to 140 GO biological process terms (shown in Table S3). Based on the corrected *P* value, the top 20 terms in biological processes were significantly related to “protein metabolic process” (GO:0019538), “cellular response to oxidative stress” (GO:0034599), “regulation of cell death” (GO:0010941), etc. (shown in Figure 3).

**Figure 4:**
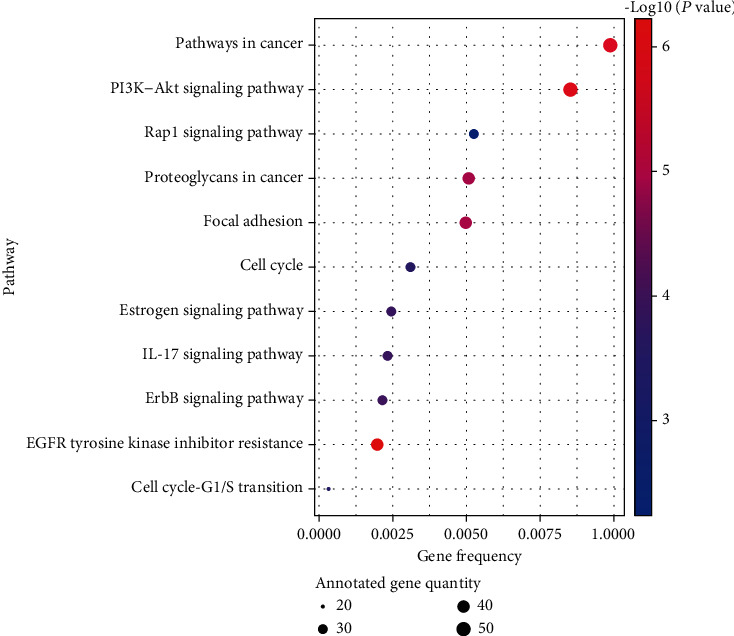
KRGG pathway analysis of target genes.

To further uncover the potential pharmacological mechanisms of kaempferol against GC, pathway analysis was conducted to explore the potential pathways affected by kaempferol. Combining the pathogenesis of GC, the pathways which have no association with GC were removed. Finally, 11 remarkably enriched terms were likely to be the major pathways in the treatment of GC (shown in Table 3, Figure 4). Results demonstrated that “EGFR tyrosine kinase inhibitor resistance,” “PI3K-Akt signaling pathway,” and “pathways in cancer” were obviously enriched. The above molecular functions and biological processes were closely related to the occurrence and development of GC, which indicated that kaempferol can treat GC through multiple targets and pathways.

### 3.4. Integration of Protein-Protein Interaction Network

**Figure 5:**
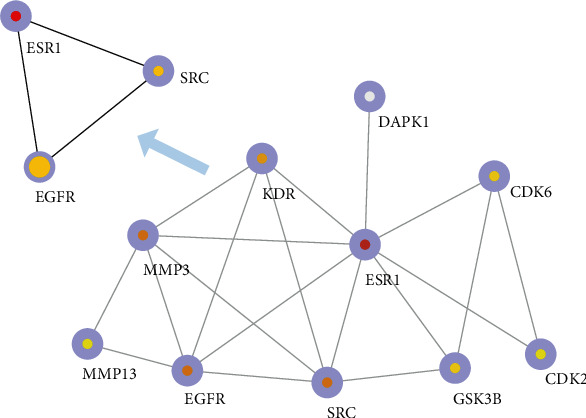
Protein-protein interaction network.

To predict potential interactions between gene candidates at the protein level, a PPI network was constructed based on the STRING database. The top 3 genes in the MCC method were chosen by the cytoHubba plugin and sequentially ordered as follows: ESR1, EGFR, and SRC. According to the interaction network diagram, ESR1, EGFR, and SRC were in the center of the network, which might play a critical role in the GC progression. As shown in Figure 5, the higher degree values are indicated by the color changes from yellow-green to red.

### 3.5. Expression Levels of the Hub Targets in GC

**Figure 6:**
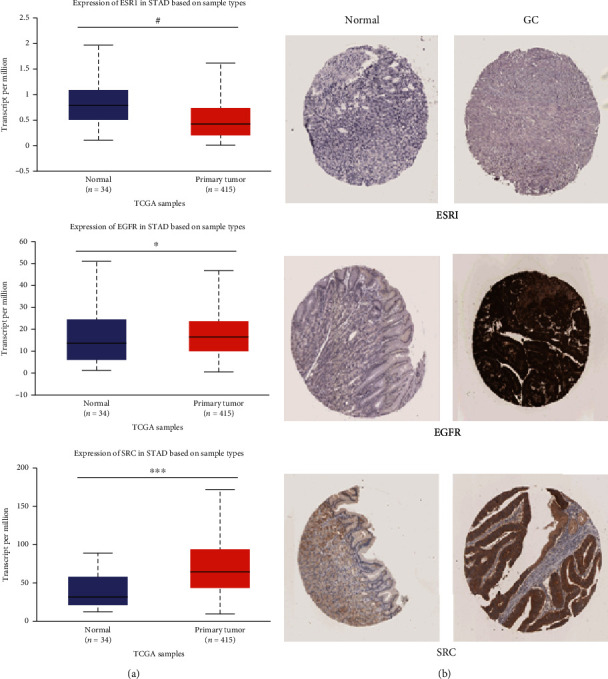
Expression levels of ESR1, EGFR, and SRC in GC and normal tissues. (a) mRNA expression of the ESR1, EGFR, and SRC gene in The Cancer Genome Atlas (TCGA) database: box plots showing the mRNA expression in GC tumors (red plot) and their normal (blue plot) tissues which were derived through UALCAN database. Data are presented as the mean ± standard error. ^#^*P* > 0.05, ^∗^*P* < 0.05, and ^∗∗∗^*P* < 0.001. (b) The representative protein expression of the ESR1, EGFR, and SRC in GC tissue and normal tissue from the immunohistochemistry data from the HPA.

The expression levels of ESR1, EGFR, and SRC in GC vs. normal tissue were obtained via the UALCAN online database. The results revealed that the mRNA expression levels of EGFR and SRC were increased in GC tissues compared with normal tissues (*P* < 0.05, Figure 6(a)). However, the mRNA expression levels of ESR1 in GC has no statistically significant difference compared with normal tissues (*P* > 0.05, Figure 6(a)).

For further validation based on the immunohistochemical data from the HPA, the protein levels of EGFR and SRC appeared positive in GC tissue samples than in normal gastric tissue samples, but the level of ESR1 in GC was negative (Figure 6(b), Table 4).

### 3.6. Survival Analysis of the Hub Genes

**Figure 7:**
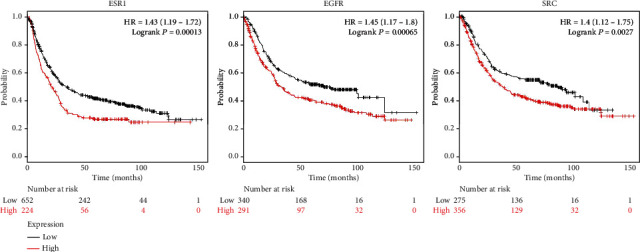
The prognostic value of the expression of the 3 hub genes. The survival data were analyzed by the Kaplan-Meier Plotter database (*P* < 0.05). Patients with expression above the median are indicated in the red line, while the black line represents expression below the median. HR represents the hazard ratio.

To further investigate whether ESR1, EGFR, and SRC contributed to the prognosis in patients, the Kaplan-Meier survival plot was used to analyze the disease-free survival of these hub genes in GC. According to the low and high expression, the OS for ESR1, EGFR, and SRC was obtained. The result showed that the prognostic value of these hub genes with high mRNA expression was associated with a poor OS for GC patients (Figure 7, Table 5). The result indicated that the expression levels of the ESR1, EGFR, and SRC were significantly associated with the clinical prognosis of GC, and those genes may play vital roles in the pathogenesis of GC.

## 4. Discussion

Network pharmacology is an emerging discipline based on the theory of systems biology. To elucidate the potential mechanism of kaempferol on GC, a network pharmacology approach that integrated drug-likeness evaluation, target identification, pathway and GO analysis, and PPI analysis was used in this work. Kaempferol is a flavonoid found in many herbs. Based on target prediction and molecular docking, 10 targets including GSK3B, DAPK1, CDK6, CDK2, EGFR, SRC, KDR, MMP13, MMP3, and ESR1 which have a good combination with kaempferol were selected as candidate targets for kaempferol against GC.

To elucidated the biological effects of kaempferol on GC, 10 targets were assigned to 140 GO biological process terms and 11 pathways. According to the GO category analysis, 10 targets were mainly involved in cell metabolism and cell apoptosis. With the emergency of metabolomics, the relationship between metabolic regulation and cancer has attracted an increasing interest. It is well-acknowledged that metabolic reprogramming is one of the hallmarks of cancer and intricately linked to oncogenesis [28]. Accumulated evidence indicates that there are various metabolic changes during the development of GC, including glucose metabolism, amino acid metabolism, lipid metabolism, and nucleotide metabolism [29, 30]. The PI3k-Akt signaling pathway, one of the enriched KEGG pathways, has a role in many cellular processes including metabolism, cell survival, motility, and cancer progression [31]. Studies have demonstrated that PI3K-Akt signaling is able to regulate nutrient transporters and metabolic enzymes and control the transcription factors that regulate the expression of key components of metabolic pathways [32, 33]. Moreover, several studies have reported that PI3K-Akt signaling can regulate the metabolism pathways involved in cellular oxidative stress, as well as aerobic glycolysis [34, 35]. Thus, targeting the PI3K-Akt signaling pathway can be an efficient avenue for the therapeutic intervention of GC. Some scholars have confirmed that kaempferol has a negative regulation on oxidative stress [36] and tumor glycolysis [37] and can suppress GC through inactivating the PI3K-Akt pathway [38]. However, whether kaempferol could regulate the metabolism of GC via the PI3K-Akt signaling pathway still needs further validation.

It is well known that one of the most fundamental traits of cancer cells is the abnormal changes in programmed cell death, namely, apoptosis [39]. The abnormal apoptosis is closely related to the formation of gastrointestinal malignancies [39]. It has been demonstrated that apoptosis plays a vital role in the morphogenesis of GC [40]. According to the KEGG pathway enrichment, PI3K-Akt signaling pathway, ErbB signaling pathway, cell cycle, and cell cycle-G1/S transition are closely related to apoptosis in GC. Previous studies have proven that the PI3K-Akt signaling pathway can suppress apoptosis and implement cell proliferation and metastasis of cancer [41]. ErbB, a member of the epidermal growth factor receptor family, has been demonstrated to interact with the regulation of cellular proliferation, differentiation, and apoptosis that promotes cell survival [42]. As an estrogen-related receptor *α* (ERR*α*) inverse agonist, kaempferol is able to increase apoptosis of cancer cells via PI3K-Akt signaling pathway and ErbB signaling pathway [38, 43, 44]. Besides, studies have confirmed that kaempferol can induce cell cycle arrest and inhibit G1/S cell cycle transition [45, 46]. Therefore, modulating the apoptosis of gastric mucosal epithelial cells may be an important way for kaempferol to treat GC.

Based on the PPI network, the top three hub genes were identified, namely, ESR1, EGFR, and SRC. The mRNA and protein expression levels of EGFR and SRC were obviously higher in GC tissues. Next, survival analysis showed that high expression of ESR1, EGFR, and SRC was related to poor OS in GC patients. Estrogen regulates cell growth and differentiation by combining its nuclear hormone receptor subtypes, ESR1 and ESR2. A clinical study has demonstrated that the expression of ESR1 is associated with poorer overall survival in patients with GC [47], while kaempferol can inhibit cell proliferation by suppressing the level of ESR1 [48], which is consistent with the predicted result of this study. EGFR, one of the members of the ErbB family of tyrosine kinase receptors, can lead to the activation of the PI3K-Akt signaling pathway. Mechanism studies have revealed kaempferol has a direct effect on EGFR activity along with the inhibition of EGFR [49]. SRC, a serine/threonine kinase, is commonly overexpressed or activated during GC development [50, 51]. Activated SRC can regulate cell proliferation, angiogenesis, adhesion, invasion, and metastasis by transducing the PI3K pathway [52]. Thus, more and more research has renewed interest in developing SRC inhibitors. A related analysis has found that kaempferol can act as a safety anticancer reagent by inhibiting the SRC [44]. Taken together, ESR1, EGFR, and SRC are crucial in the pathogenesis of GC. These targets may be the key points of the therapeutic action of kaempferol in GC.

## 5. Conclusions

Kaempferol is a promising compound, which is expected to be developed as a safe and effective multitarget drug against GC. Our network pharmacological analysis predicted that kaempferol may exert an anti-GC effect through multiple targets, pathways, and biological processes, thereby regulating the cell metabolism and cell apoptosis. Moreover, this effect could be related to the inhibition of ESR1, EGFR, and SRC by kaempferol. Further verification studies are required to confirm the clinical efficacy of kaempferol and its mechanisms against GC.

## Figures and Tables

**Table 1:** The information of 10 targets.

Target	Common name	UniProt ID	ChEMBL ID	Target class
Glycogen synthase kinase-3 beta	GSK3B	P49841	CHEMBL262	Kinase
Death-associated protein kinase 1	DAPK1	P53355	CHEMBL2558	Kinase
Cyclin-dependent kinase 6	CDK6	Q00534	CHEMBL2508	Kinase
Cyclin-dependent kinase 2	CDK2	P24941	CHEMBL301	Kinase
Epidermal growth factor receptor ErbB1	EGFR	P00533	CHEMBL203	Kinase
Tyrosine-protein kinase SRC	SRC	P12931	CHEMBL267	Kinase
Vascular endothelial growth factor receptor 2	KDR	P35968	CHEMBL279	Kinase
Matrix metalloproteinase 13	MMP13	P45452	CHEMBL280	Protease
Matrix metalloproteinase 3	MMP3	P08254	CHEMBL283	Protease
Estrogen receptor alpha	ESR1	P03372	CHEMBL206	Nuclear receptor

**Table 2:** Vina scores and cavity information of the docking simulation pose for each targeted protein and kaempferol.

Receptors	PDB ID	Vina score	Cavity size	Center			Size		
				*x*	*y*	*z*	*x*	*y*	*z*
GSK3B	1h8f	-9.1	650	22	2	21	21	21	21
DAPK1	5aux	-8.7	903	-19	1	-10	35	21	21
CDK6	4aua	-9.7	1253	25	38	-2	21	21	31
CDK2	4ek3	-8.1	1220	21	24	24	30	21	21
EGFR	6s9b	-7.9	1392	-54	30	-1	35	35	21
SRC	1yoj	-8.1	751	-4	18	15	21	21	21
KDR	3vid	-8.2	770	-4	11	-33	21	29	21
MMP13	5uwl	-8.9	1269	48	-18	11	33	28	31
MMP3	1b3d	-9.6	750	-19	30	7	21	21	28
ESR1	3os8	-8.8	1436	16	37	-67	35	21	21

**Table 3:** 11 representative pathways according to gene count.

Pathway ID	Pathway	Corrected *P* value	Gene count	Annotated genes
KEGG:hsa01521	EGFR tyrosine kinase inhibitor resistance	7.46*E*-07	4	EGFR, GSK3B, KDR, SRC
KEGG:hsa04151	PI3K-Akt signaling pathway	2.51*E*-06	5	CDK2, CDK6, EGFR, GSK3B, KDR
KEGG:hsa05200	Pathways in cancer	5.22*E*-06	5	CDK2, CDK6, DAPK1, EGFR, GSK3B
KEGG:hsa04510	Focal adhesion	3.11*E*-05	4	EGFR, GSK3B, KDR, SRC
KEGG:hsa05205	Proteoglycans in cancer	3.36*E*-05	4	EGFR, ESR1, KDR, SRC
KEGG:hsa04012	ErbB signaling pathway	3.14*E*-04	3	EGFR, GSK3B, SRC
KEGG:hsa04657	IL-17 signaling pathway	3.98*E*-04	3	GSK3B, MMP13, MMP3
KEGG:hsa04915	Estrogen signaling pathway	4.66*E*-04	3	EGFR, ESR1, SRC
KEGG:hsa04110	Cell cycle	9.46*E*-04	3	CDK2, CDK6, GSK3B
KEGG:hsa_M00692	Cell cycle-G1/S transition	0.00131	2	CDK2, CDK6
KEGG:hsa04015	Rap1 signaling pathway	0.0046	3	EGFR, KDR, SRC

**Table 4:** Immunohistochemistry analysis of the ESR1, EGFR, and SRC in GC and normal tissues.

Gene	Patient ID	Type	Age	Sex	Intensity	Quantity	Location
ESR1	328	Normal tissue	75	Female	Negative	None	None
	2626	Adenocarcinoma	79	Female	Negative	None	None
EGFR	2411	Normal tissue	71	Female	Negative	None	None
	664	Adenocarcinoma	50	Female	Strong	>75%	Cytoplasmic/membranous
SRC	2130	Normal tissue	56	Female	Weak	75%-25%	Cytoplasmic/membranous
	2378	Adenocarcinoma	59	Male	Strong	>75%	Cytoplasmic/membranous

**Table 5:** Median survival of ESR1, EGFR, and SRC.

Gene	Low expression cohort (months)	High expression cohort (months)
ESR1	35.2	22
EGFR	76.2	33.6
SRC	85.8	36.4

## Data Availability

The data used to support the findings of this study are available from the corresponding authors upon request.

## Abbreviations

GC: Gastric cancer
OMIM: Online Mendelian Inheritance in Man
TTD: Therapeutic Target Database
PPI: Protein-protein interaction
OS: Overall survival
GO: Gene Ontology
KEGG: Kyoto Encyclopedia of Genes and Genomes.

## Supplementary Materials

Supplementary Materials Table S1: information on kaempferol-related targets. Table S2: information on GC-related targets. Table S3: GO terms of therapy target genes and their corresponding count, corrected *P* values, and gene count.
